# Clonidine versus captopril for treatment of postpartum very high blood pressure: study protocol for a randomized controlled trial (CLONCAP)

**DOI:** 10.1186/1742-4755-10-37

**Published:** 2013-07-30

**Authors:** Carlos Noronha-Neto, Leila Katz, Isabela C Coutinho, Sabina B Maia, Alex Sandro Rolland Souza, Melania Maria Ramos Amorim

**Affiliations:** 1 Obstetric Intensive Care Unit, Instituto de Medicina Integral Prof. Fernando Figueira, Recife, PE, Brazil; 2 Department of Obstetrics and Gynecology, Federal University of Campina Grande, Campina Grande, PB, Brazil

**Keywords:** Severe preeclampsia, Hypertension, Pregnancy, Postpartum, Very high blood pressure, Randomized controlled trial, Antihypertensive agents

## Abstract

**Background:**

The behavior of arterial blood pressure in postpartum of women with hypertension and pregnancy and the best treatment for very high blood pressure in this period still need evidence. The Cochrane systematic review assessing prevention and treatment of postpartum hypertension found only two trials (120 patients) comparing hydralazine with nifedipine and labetalol for the treatment of severe hypertension and did not find enough evidence to know how best to treat women with hypertension after birth. Although studies have demonstrated the effectiveness of treatment with captopril, side effects were reported. Because of these findings, new classes of antihypertensive drugs began to be administered as an alternative therapy. Data on the role of clonidine in this particular group of patients, its effects in the short and long term are still scarce in the literature.

**Objectives:**

To determine the effectiveness of clonidine, compared to captopril, for the treatment of postpartum very high blood pressure in women with hypertension in pregnancy.

**Methods/design:**

The study is a triple blind randomized controlled trial including postpartum women with diagnosis of hypertension in pregnancy presenting very high blood pressure, and exclusion criteria will be presence of heart disease, smoking, use of illicit drugs, any contraindication to the use of captopril or clonidine and inability to receive oral medications.

Eligible patients will be invited to participate and those who agree will be included in the study and receive captopril or clonidine according to a random list of numbers. The subjects will receive the study medication every 20 minutes until blood pressure is over 170 mmHg of systolic blood pressure and 110 mmHg diastolic blood pressure. A maximum of six pills a day for very high blood pressure will be administered. In case of persistent high blood pressure levels, other antihypertensive agents will be used.

During the study the women will be subject to strict control of blood pressure and urine output. This proposal has already obtained approval of the local Institutional Review Board of the coordinating center (IMIP, Recife, Brazil) and of the National Council for Ethics in Research (CONEP) of the Brazilian Ministry of Health.

**Trial registration:**

Clinical Trials Register under the number NCT01761916.

## Introduction

Hypertension complicates approximately 10% of pregnancies [1] and is the leading cause of maternal death in Brazil [2] and the third leading cause in the world [3]. It also entails increased maternal morbidity associated with complications such as eclampsia, HELLP syndrome, hemorrhage, pulmonary edema, renal failure and coma [4]. Perinatal morbidity and mortality are also increased [5]. Much has been studied about the repercussions of hypertension for the mother and the fetus, but there are relatively few studies evaluating the behavior of postpartum blood pressure, its impact and what is the better pharmacological treatment during this period [6-11].

Although the true prevalence of postpartum hypertension is unknown, it is important to monitor blood pressure levels after delivery, because they can continue to increase in women with previous diagnosis of hypertension or hypertensive crisis can develop only postpartum [6]. According to a recent review of the etiology and management of postpartum hypertension-preeclampsia the reported prevalence of new onset postpartum hypertension or preeclampsia ranges from 0.3–27.5% [6]. Complications of severe hypertension include stroke, eclampsia, congestive heart failure, acute pulmonary edema, renal failure and death [4,12]. One study demonstrated that 44% of eclampsia occurs in the postpartum period, usually in the first 48 hours [13].

It has been shown that blood pressure increases progressively over the first five days after birth, peaking on three to six days after delivery [14] It is believed that this pattern of blood pressure results from the mobilization from the extravascular to intravascular space of six to eight liters of total body water and the and the 950 mEq of total body sodium accumulated during pregnancy. The urinary sodium excretion was observed on days 3–5 postpartum [15], and it was postulated to result from an increase in atrial natriuretic peptide (ANP). ANP have roles in natriuresis, inhibition of aldosterone and angiotensin II and vasopressin [16] and was found to rise during the first week after delivery [17].

Hypertension, proteinuria and biochemical changes caused by pre-eclampsia may persist for six to 12 weeks postpartum [18]. Although it has been postulated that the exposure time could be short to cause target organ damage, the presence of kidney disorders and persistence of hypertension in subsequent evaluations have been demonstrated, which generated doubts about the best management in this period [19].

The effectiveness of different antihypertensive medications in the postpartum period has not been adequately assessed. In the Cochrane systematic review specifically evaluating prevention and treatment of postpartum hypertension, nine trials were included, four of those evaluating prevention furosemide, nifedipine and L-arginine compared to placebo, three studies evaluated timolol, oral hydralazine, or oral nifedipine compared with methyldopa and only two studies (120 patients) evaluated the treatment of severe hypertension with intravenous hydralazine, labetalol intravenous or sublingual nifedipine. In these last studies, there were no maternal deaths or hypotension. Use of additional antihypertensive therapy did not differ between groups (RR 0.58, 95% CI 0.04 to 9.07; two trials), but the trials were not consistent in their effects. The reviewers conclude that there are no reliable data to guide management of women who are hypertensive postpartum. Any antihypertensive agent used should be based on a clinician's familiarity with the drug [9]. In the setting of postpartum hypertensive crisis there is no consensus, although recently experts have recommended intravenous labetalol or hydralazine if there is persistent elevations in blood pressure (BP) to levels ≥160 mmHg systolic and/or ≥ 110 mmHg diastolic [6].

Captopril is an inhibitor of angiotensin I converting enzyme and is used for treating hypertensive crisis [20]. Although the use of captopril during pregnancy is contraindicated [21], its use after delivery has been described [22] and the drug is compatible with lactation [23,24]. However, side effects have been reported such as dry cough, hypotension and decreased renal function [24].

The use of captopril postpartum was tested in an open clinical trial with 79 patients with a diagnosis of pre-eclampsia and eclampsia. Of these, 71 received captopril at a dose of 25 mg at intervals of 30 minutes up to three doses and the remaining eight received hydralazine intravenously at a dose of 5 mg, maximum of four doses with an interval of 20 minutes. Of the mothers who received captopril, 52 (73.3%) responded satisfactorily to the drug with a reduction of up to 25% of blood pressure levels. Regarding the number of doses administered, 20 patients (38%) required one dose, 26 (50%) two doses and six (11%), three doses. No adverse maternal effects were observed. There were also no changes in diuresis and creatinine levels in the neonate. Given these findings, the authors recommend the use of captopril for the management of hypertensive crisis in the puerperium [22]. Low levels of captopril are found in breast milk with small amounts of the drug ingested by the infant. So, it would not be expected any adverse effects in breastfed infants [23-25]. Notwithstanding, no randomized controlled trials (RCT) were found comparing captopril with other drugs or placebo in postpartum hypertensive women.

Clonidine belongs to the class of alpha-2-agonist with central action [26] and began to be used in postpartum women with some restriction on the use of ACE inhibitors and its hypotensive effect for peak pressure was satisfactory [27]. The drug is compatible with lactation and no typical clonidine side effects were seen in infants whose mothers were taking clonidine [28,29]. What is not known yet is how long clonidine lowers blood pressure and what is the duration of its effect compared to captopril.

There were no reports in the database searched (Embase, Scopus, Cochrane Library, Lilacs and Pubmed), neither randomized controlled trials that prove the effectiveness of clonidine for the treatment of hypertensive crisis in this particular group of patients or studies comparing this drug with captopril.

The objective of this study is to determine the effectiveness of clonidine compared to captopril for treatment of very high blood pressure in postpartum women in a randomized controlled trial.

### Objectives and hypothesis

The overall objective is to determine the effectiveness of clonidine compared to captopril, for treatment of postpartum women with very high blood pressure.

#### Specific objectives

Analyzing postpartum patients with very high blood pressure randomized to receive clonidine or captopril, the specific objectives are to compare:

#### Primary outcome

• Mean arterial blood pressure

#### Secondary outcomes

• Need of another antihypertensive agent for very high blood pressure;

• Need to maintain the antihypertensive therapy;

• Daily mean systolic blood pressure

• Daily mean diastolic blood pressure

• Number of days with hypertensive peaks

• Daily mean of hypertensive peaks

• Number of days until blood pressure control

• Number of days using antihypertensive drugs for treatment of hypertensive peaks

• Number of antihypertensive drugs associated

• Need for sodium nitroprusside to control hypertension

• Number of days of obstetric ICU stay.

### Main hypothesis

In postpartum patients with postpartum very high blood pressure randomized to receive clonidine compared to the women receiving captopril:

• Mean blood pressure is lower

• Need of other antihypertensive agents for very high blood pressure is lower

• Need to maintain other antihypertensive agents is lower

• Daily mean systolic blood pressure is lower

• Daily mean diastolic blood pressure is lower

• Number of days with hypertensive peaks is lower

• Number of days until blood pressure control is lower

• Number of days using antihypertensive drugs for treatment of hypertensive peaks is lower

• Need of association with other antihypertensive drug for treatment of hypertensive peaks is lower

• Number of antihypertensive drugs associated is lower

• Need for sodium nitroprusside to control hypertension is lower

• Number of days of obstetric ICU stay is lower.

## Methods/design

### Study design

The present study is a triple blind randomized controlled trial. The SPIRIT guidelines were used to prepare this protocol [30].

### Study population and location

The study population will include all eligible women with hypertension in pregnancy admitted to the obstetrical ICU in the postpartum period.

### Eligibility criteria

The inclusion criterion is the presence of very high blood pressure in postpartum women with hypertension in pregnancy receiving magnesium sulfate for prevention or treatment of eclampsia (which includes both severe and superimposed preeclampsia or eclampsia cases). Exclusion criteria are: other associated maternal diseases (such as diabetes mellitus, collagen vascular disease, glomerulonephritis, lung and heart disease); smoking; use of any antihypertensive agent for very high blood pressure before admission to ICU or illicit drugs that may interfere with maternal hemodynamics; contraindications to the use of captopril (acute kidney injury, chronic liver disease and hypersensitivity to the drug) and contraindications to the use of clonidine (sick sinus syndrome, chronic liver disease and hypersensitivity to the drug).

### Procedures for selecting participants and randomization

Eligible patients will be invited to participate and those who agree will be included in the study and receive clonidine or captopril according to a random list of numbers generated by the Random Allocation Software (Ispharan Iran), version 1.0. This list of randomization will be provided by the statistician to the pharmacist who will be responsible for preparing the packages containing either the clonidine or captopril, both in an identical presentation, with the identification number of list labeled. This procedure will be followed in order to guarantee the concealment of allocation of patients in both arms. Considering both drugs have rare identifiable immediate effects, either patients and medical staff should be blind of the intervention condition in each case. Study medication, after delivered by the pharmaceutical industry to the pharmacy of the coordinating center, will be packed as previously described and according the random list. The drugs will be kept at the ICU under the responsibility of the nurse, and when included the patient will receive the drug in the package corresponding to the inclusion. The subjects will receive the study medication at the moment of the inclusion (very high blood pressure) and the drug will be repeated after 20 minutes if the blood pressure levels have not responded to the administered drug, to a maximum of six doses. Other episodes of very high blood pressure during the day will be managed likewise, respecting the maximum of six doses of the study medication a day.

If attending physicians observe that the blood pressure levels do not improve or if the 6 doses of medication for very high blood pressure where used, another drug for very high blood pressures (hydralazine or sodium nitroprusside) will be used, according to the physicians’ choice and judgment. During the study period other anti hypertensive agents used for maintenance may be initiated according to the physicians’ choice and judgment.

During the observation period the women will be subject to the ICU routine which includes strict control of blood pressure and urine output (diuresis evaluated spontaneous or urinary catheter). Laboratory tests will be performed at regular intervals of 24 hours (blood count, coagulation, renal and hepatic function).

### Sample size calculation

To calculate the sample size, as there were no studies evaluating the use of clonidine for the treatment of hypertensive crisis in the puerperium, nor a comparison with captopril for this purpose, we conducted a pilot study with 30 postpartum women with hypertensive disorders, 15 of each group (captopril vs. clonidine) in order to obtain the most suitable calculation of the sample.

After completion of the pilot study and through statistical calculator Open Epi 3.01 (CDC, GA), the daily mean of hypertensive peaks in each group (called A and B for this purpose) was used to calculate the sample with a power of 90% and a confidence level of 95%. Considering a mean of 2.8 hypertensive peaks/day in group A, with a standard deviation of 2.0 and a mean of 6.2 hypertensive peaks/day with a standard deviation of 6.2 in group B, a total of 90 patients was estimated already predicting eventual losses or differences between the groups (original sample size of 78 women).

### Daily mean of hypertensive peaks

#### Variables

##### Independent variable

• Use of clonidine or captopril

#### Dependent variables

Mean arterial blood pressure, need of another antihypertensive agent for very high blood pressure, need to maintain the antihypertensive therapy, daily mean systolic blood pressure, daily mean diastolic blood pressure, number of days with hypertensive peaks, number of days until blood pressure control (absence of episodes of very high blood pressure), number of days using antihypertensive drugs for treatment of hypertensive peaks, need of association with other antihypertensive drugs for treatment of hypertensive peaks, number of antihypertensive drugs associated, need for sodium nitroprusside to control hypertension and number of days of obstetric ICU stay.

### Main outcomes

• Hypertensive disorders of pregnancy: defined according to the National High Blood Pressure Education Program Working Group on High Blood Pressure in Pregnancy [18].

• Very high blood pressure episode: systolic blood pressure (SBP) ≥ 180 mmHg and/or diastolic blood pressure (DBP) ≥ 110 mmHg [18].

• Control of arterial blood pressure: absence of episodes of very high blood pressure.

#### Data collection procedures

##### Data collection

The data will be entered in a specific database created in the public domain statistical program Epi-Info version 7 for Windows vista. The data entry will be made after reviewing the forms. Every month this database will be reviewed by the researcher, obtaining listing of variables and correcting any inconsistencies or missing information from the query to the forms. Data missing in medical charts should be collected from other sources, such as hospital's database, prenatal cards, transference documents and others. Tests of consistency and frequency distribution tables of the main variables will be obtained to correct any errors. In the case of finding inconsistencies or lack of information, the forms will be consulted. At the end of typing, listings for the final correction will again be obtained with creation of the definitive database, which will be submitted to the tests of cleaning and consistency of information and hence statistical analysis will be performed.

### Data analysis plan

**Figure 1:**
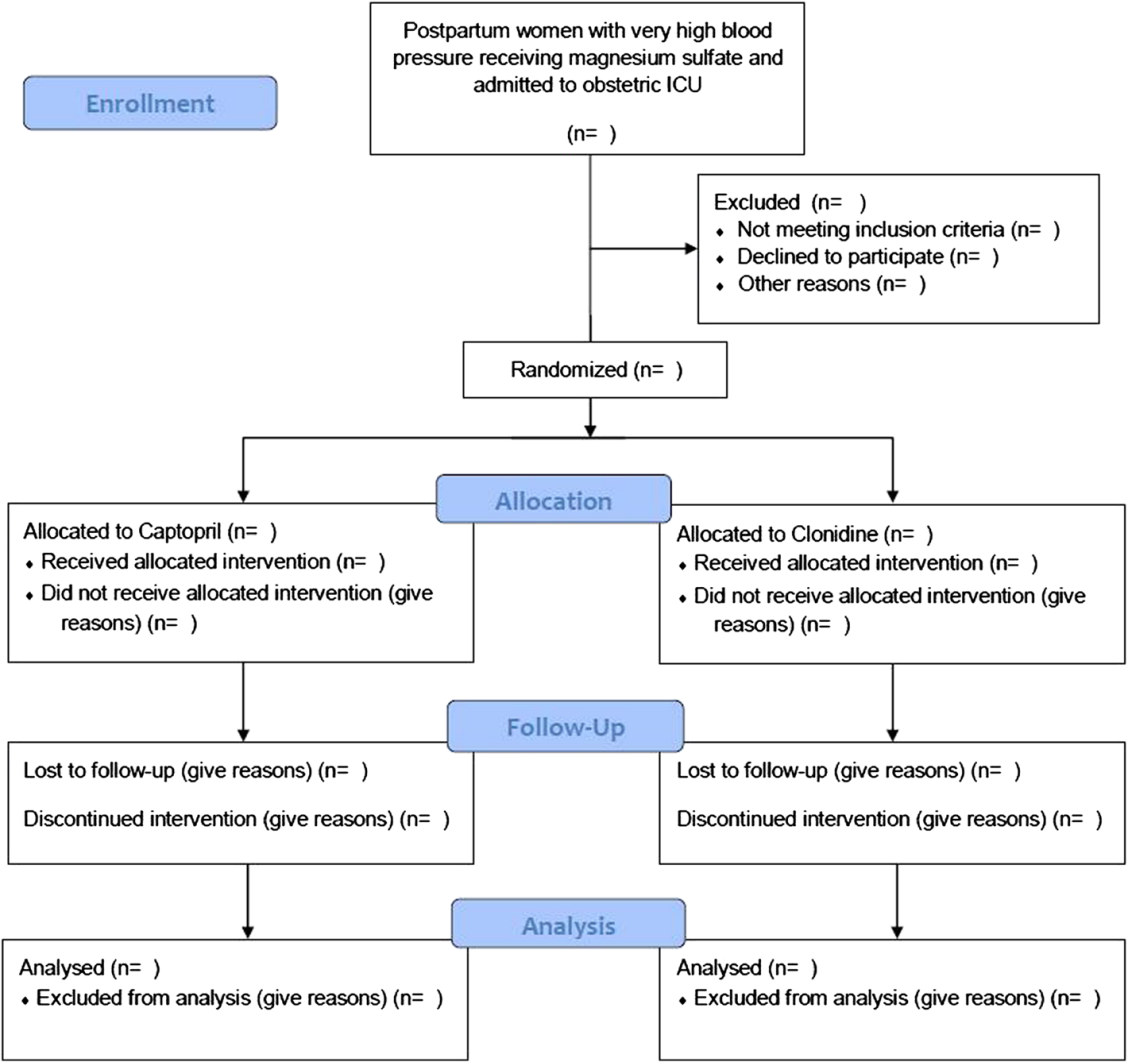
**Study design and population (CONSORT, 2010)**[31]**.**

The data analysis will be performed using the public domain software Epi Info version 7 (Centers for Disease Control and Prevention, Atlanta, GA), or the newest available version under the intention to treat principle. The statistician and the investigators will remain blind to the treatment groups until the tables will be prepared and the analysis concluded. The approach for analysis will be that showed in Figure 1 using an intention-to-treat strategy and following the correspondent recommendations from the CONSORT statement [31]. The characteristics of the participants in each group will be compared with Student’s *t* test for continuous variables with normal distribution and Mann–Whitney U test for discrete and ordinal variables or those with non-normal distribution. Repeated measures analysis of variance (ANOVA) will be used for comparing daily mean blood pressure in both groups. Categorical variables will be compared with Pearson’s χ [2] test or Fisher’s exact test, as appropriate. P values for all tests will be two tailed at a 5% level of significance. Risk ratios and their 95% confidence intervals will be calculated as a measure of relative risk. The number needed to treat (NNT) and its 95% confidence interval will be calculated for the outcomes in which a beneficial effect of dexamethasone treatment is achieved, using the EBM calculator [http://moosenose.com/EBCalculator.htm]; in case of adverse effects the number needed to harm (NNH) and its 95% confidence will also be calculated.

### Quality control

The local researchers will maintain a record of problems occurred during the study and any doubt should be solved with the project's coordinators and the Steering Committee.

### Ethical issues

The original protocol of this research proposal has already obtained approval of the local Institutional Review Board from the coordinating center (IMIP, Recife, Brazil), and of the National Committee for Ethics in Research (CONEP) of the Brazilian Ministry of Health, under the number 3079–12. The protocol also was published in the Clinical Trials Register under the number NCT01761916 (http://clinicaltrials.gov/ct2/show/NCT01761916?term=CLON+CAP&rank=1). Postpartum patients with hypertension and very high blood pressure will only be included if they agree to participate and sign the informed consent. All principles related to research in human beings established by the Brazilian National Health Council according to the Declaration of Helsinki will be followed. The confidentiality on women's data and medical care will be ensured regardless of whether they participate in the study or not.

## Discussion

### Technical and scientific contributions of the study

Postpartum hypertension is still an understudied theme and may represent either the continuation of a pre-existing hypertension and new onset hypertension after delivery. Evidence of good quality evaluating the treatment of hypertensive crisis during this period are not yet available. Very high blood pressure may be associated with increased risk of stroke, eclampsia, congestive heart failure, acute pulmonary edema, renal failure and other morbidities, but the best drug for its treatment in the postpartum period remains to be established. Both captopril and clonidine have been proposed for this purpose, being both therapeutic options widely available, inexpensive and compatible with lactation. However, their effectiveness and safety have not been demonstrated in randomized controlled trials. This study is important because it can bring substantial information on antihypertensive treatment in the postpartum period with affordable medicines which are available in poor countries, especially those where hypertension remains a major cause of maternal death and labetalol is not available. The study may also determine which antihypertensive drug is safer and more effective for treatment of postpartum very high blood pressure, which can reduce maternal complications and length of hospital stay.

## Abbreviations

ICU: Intensive care unit; SBP: Systolic blood pressure; DBP: Diastolic blood pressure; IRB: Institutional review board; NNH: Number needed to harm; NNT: Number needed to treat; RCT: Randomized controlled trials.

## Competing interests

The authors declare that they have no competing interests.

## Authors’ contributions

The first version of this protocol was drafted by MA and LK. All authors have made substantive intellectual contributions to the manuscript and read and approved its final version.
